# Incidental Dysembryoplastic Neuroepithelial Tumor Identified During the Evaluation of Acute Headache and Vomiting in a Pediatric Patient

**DOI:** 10.7759/cureus.107151

**Published:** 2026-04-16

**Authors:** Bosaina Otour, Sania Shahid, Ahmad AlHammada, Sana Ahmed, Rokia Malahifci

**Affiliations:** 1 Pediatric Emergency Medicine, Al Jalila Children's Specialty Hospital, Dubai, ARE; 2 Pediatric Emergency Medicine, Dubai Academic Health Corporation, Dubai, ARE; 3 General Pediatrics, American Hospital, Dubai, ARE; 4 Medicine, Dubai Medical University, College of Medicine, Dubai, ARE; 5 Internal Medicine, Dubai Medical University, College of Medicine, Dubai, ARE

**Keywords:** benign brain tumor, dysembryoplastic neuroepithelial tumour (dnet), headache, pediatric emergency department (ped), vomiting pediatric

## Abstract

Acute headache in pediatric patients often warrants neuroimaging when accompanied by vomiting or concerning clinical features. While most cases are benign or infectious in etiology, incidental structural abnormalities may be discovered. Dysembryoplastic neuroepithelial tumors (DNETs) are rare, benign, WHO grade I glioneuronal tumors, commonly associated with epilepsy and typically located in cortical regions.

We present the case of an eight-year-old boy who presented with acute headache and vomiting and was found to have a left frontal lobe cystic lesion with a “soap bubble” appearance on magnetic resonance imaging (MRI), suggestive of DNET. The lesion was incidentally discovered during evaluation for headache in the context of group A streptococcal (GAS) pharyngitis. The patient had no seizure history or focal neurological deficits. Multidisciplinary consensus favored conservative management with serial imaging. This case highlights the importance of careful interpretation of neuroimaging findings in pediatric headache and supports a watchful-waiting strategy in asymptomatic patients with imaging features consistent with DNET.

## Introduction

Headache is a common presenting complaint in pediatric emergency departments and is most often attributable to benign causes such as viral illness, dehydration, febrile illness, sinusitis, and primary headache disorders, including migraine and tension-type headaches [1]. In most cases, these are self-limiting and do not require extensive investigation. However, the presence of red-flag features such as persistent or early morning vomiting, progressively worsening headache, nocturnal awakening, altered mental status, seizures, or focal neurological deficits (e.g., weakness, visual disturbances, and cranial nerve abnormalities), or signs of raised intracranial pressure may warrant further evaluation with neuroimaging to exclude serious intracranial pathology [2].

Dysembryoplastic neuroepithelial tumors (DNETs) are rare, benign glioneuronal tumors, classified as WHO grade I lesions [3]. They are most commonly diagnosed in children and young adults and are classically associated with drug-resistant epilepsy [4]. Imaging typically demonstrates a cortical-based lesion with a multinodular or “soap-bubble” appearance and minimal mass effect [5].

Incidental identification of DNET in patients without seizures is uncommon. We report a pediatric case in which a lesion suggestive of DNET was identified during evaluation of acute headache and vomiting secondary to group A streptococcal (GAS) pharyngitis. This case highlights the importance of recognizing red-flag symptoms, correlating clinical findings with imaging, and carefully interpreting incidental neuroimaging abnormalities, while maintaining a broad differential diagnosis in pediatric patients presenting with headache.

## Case presentation

An eight-year-old, previously healthy boy presented to the emergency department with a two-day history of sore throat, nasal congestion, headache, and vomiting. He was initially evaluated for an upper respiratory tract infection. Rapid antigen testing was positive for GAS, and inflammatory markers were elevated (C-reactive protein 80 mg/L; procalcitonin 0.42 ng/mL), as shown in Table 1.

**Table 1:** Laboratory investigations PCOT: point of care testing; PCR: polymerase chain reaction

Laboratory Parameter	Result	Reference Range
Inflammatory Markers		
C-reactive protein (CRP)	80.8 mg/L (High)	0.4 - 1.3 mg/L
Procalcitonin	0.42 mg/L (High)	<0.05 ng/mL
Complete Blood Count		
WBC count	8.4 × 10^3^/µL	5.0 - 13.0 × 10^3^/µL
RBC count	5.36 × 10^6^/µL (High)	4.00 - 5.20 × 10^6^/µL
Neutrophils (absolute)	6.27 × 10^3^/µL	2.0 - 8.0 × 10^3^/µL
Hemoglobin	12.7 g/dL	11.5 - 15.5 g/dL
Mean corpuscular volume (MCV)	73.3 fL (Low)	77.0 - 95.0 fL
Platelet count	325 × 10^3^/µL	170 - 450 × 10^3^/µL
Comprehensive Metabolic Panel		
Sodium	138 mmol/L	133 - 146 mmol/L
Potassium	4.4 mmol/L	4.2 - 6.0 mmol/L
Creatinine	0.42 mg/dL	0.4 - 1.0 mg/dL
Total calcium	9.8 mg/dL	8.6 - 11.0 mg/dL
Glucose	121 mg/dL	72 - 112 mg/dL
Total protein	7.6	-
Bilirubin, total	0.49	-
Group A streptococcus antigen detection (PCOT)	Positive	-
Respiratory screening panel PCR nasopharynx	Negative	-
Blood culture	Negative	-

Despite initial improvement, the headache was described as severe at onset (10/10), frontal in location, and associated with four to five episodes of non-bilious, non-bloody vomiting. Due to the severity of symptoms, further evaluation was pursued.

On examination, the patient was alert and hemodynamically stable. Neurological examination was normal, with a Glasgow Coma Scale score of 15. Cranial nerves were intact; motor and sensory examinations were normal; reflexes were symmetrical; and coordination was preserved. Fundoscopic examination revealed no papilledema.

**Figure 1:**
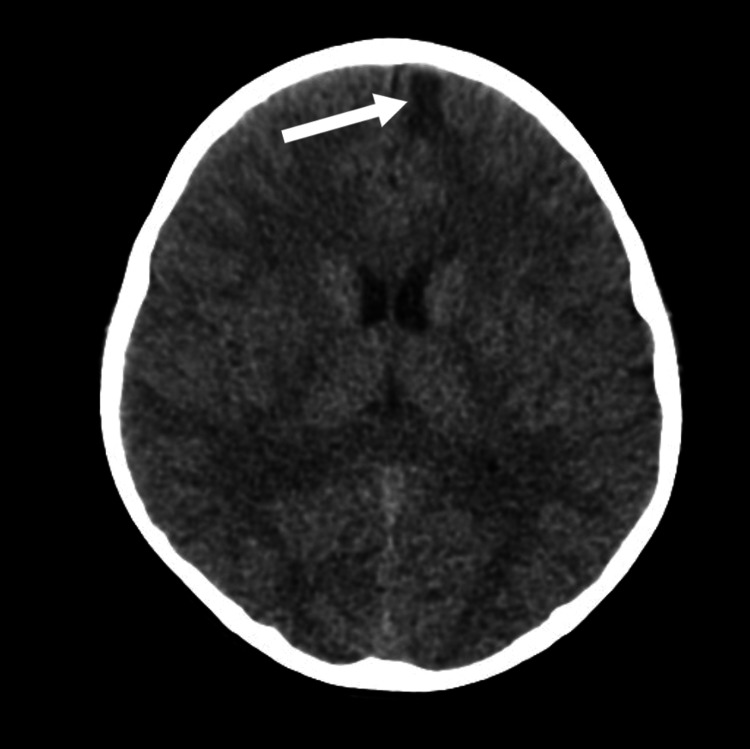
CT brain without contrast CT brain without contrast showing a small (1.8 × 1.3 cm) white matter hypodensity with relative sparing of the cortical margin, noted in the left frontal lobe (arrow). No evidence of volume loss is seen. No obvious mass effect or midline shift. CT: computed tomography

Given the severity of the headache and vomiting, neuroimaging was performed to exclude intracranial pathology. A non-contrast computed tomography (CT) scan of the brain demonstrated a small (1.8 × 1.3 cm) hypodense lesion within the left frontal white matter, with relative cortical sparing, without evidence of hemorrhage, hydrocephalus, midline shift, or significant mass effect (Figure 1). 

**Figure 2:**
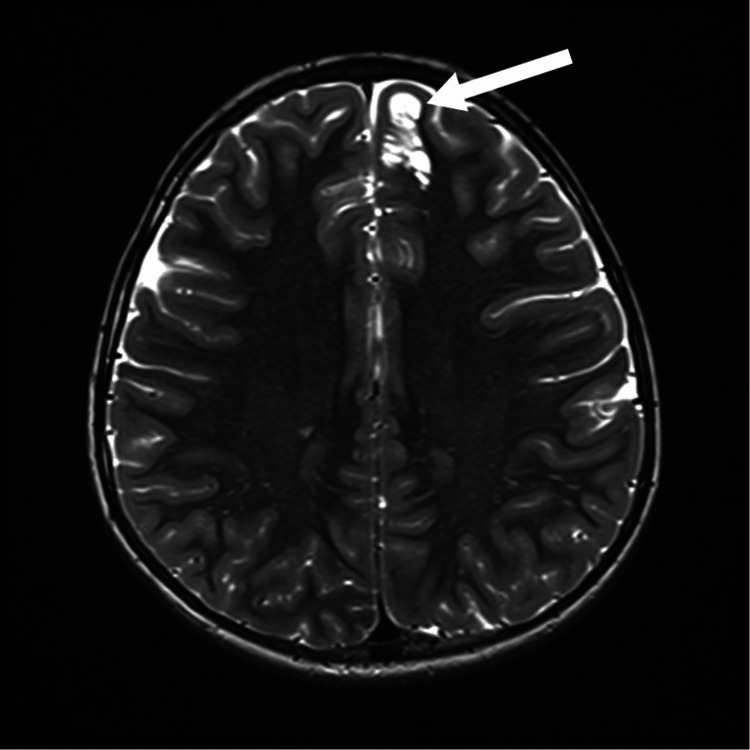
MRI of the brain The described bubbly subcortical lesion in the left frontal gyrus, with multiple septations, sparing the cortex and lacking diffusion restriction and enhancement, most likely represents a multinodular and vacuolating neuronal tumor (MVNT), a benign WHO grade 1 entity, common in pediatric patients. MRI: magnetic resonance imaging

Subsequent magnetic resonance imaging (MRI) of the brain with contrast revealed a cystic lesion in the left frontal lobe with a characteristic “soap-bubble” appearance, without diffusion restriction, enhancement, or significant perilesional edema (Figure 2). 

The radiologic findings suggested a low-grade glioneuronal tumor. A broad differential diagnosis was considered, including DNET and multinodular vacuolating neuronal tumor (MVNT), both of which can demonstrate similar imaging features. Other considerations included low-grade glioma, such as pilocytic astrocytoma; however, the absence of contrast enhancement, edema, or mass effect made this less likely. Non-neoplastic etiologies, including congenital cortical malformations and, less likely, infectious or inflammatory processes, were also considered but were not supported by the imaging pattern or clinical context.

Given the absence of seizures, focal neurological deficits, or concerning radiologic features, the lesion was considered most consistent with a benign entity. A multidisciplinary decision was made to proceed with conservative management and serial imaging surveillance.

The patient was treated for streptococcal pharyngitis and remained neurologically stable throughout hospitalization. He was discharged with outpatient follow-up arranged with pediatric neurology and neurosurgery, including repeat MRI with contrast and spectroscopy at three months.

## Discussion

DNETs are benign glioneuronal tumors strongly associated with epilepsy, particularly in pediatric populations [3,4]. They are most commonly located in the temporal lobe but may also occur in the frontal regions [5]. Radiologically, they are characterized by cortical-based, well-demarcated lesions with a multinodular architecture, minimal edema, and little to no contrast enhancement. The “soap-bubble” appearance on MRI is considered characteristic [5,6].

The differential diagnosis of a cystic, multinodular cortical lesion in pediatric patients includes several benign and low-grade entities. DNET remains a leading consideration due to its typical imaging features. However, MVNT, a benign WHO grade I lesion, may demonstrate similar radiological characteristics and is increasingly recognized as an incidental “leave-me-alone” lesion.

Low-grade gliomas, such as pilocytic astrocytoma, are also important considerations but typically demonstrate features such as contrast enhancement, a cyst-with-mural-nodule configuration, or surrounding edema - features that were absent in this case. Additionally, congenital cortical malformations, including focal cortical dysplasia, may mimic neoplastic processes but are usually associated with cortical disorganization and commonly present with seizures.

Infectious and inflammatory etiologies, such as focal encephalitis or granulomatous disease, were also considered. However, the absence of neurological deficits, systemic signs of central nervous system infection, and the well-defined, non-enhancing appearance of the lesion made these diagnoses unlikely.

In this patient, the presenting symptoms were attributable to acute streptococcal pharyngitis, supported by elevated inflammatory markers and positive microbiological testing. The intracranial lesion was therefore considered an incidental finding.

The primary clinical presentation of DNET is seizures, particularly drug-resistant epilepsy [4,7]. Although incidental discovery in non-epileptic patients is uncommon, it is increasingly reported with the widespread use of neuroimaging [8].

Management of DNET depends on symptomatology. Surgical resection is typically reserved for patients with drug-resistant epilepsy, with favorable outcomes [7]. In asymptomatic patients, or those without seizures, conservative management with serial imaging is a reasonable and widely accepted approach [9,10].

This case highlights the importance of correlating radiologic findings with clinical presentation and adopting a multidisciplinary approach to avoid unnecessary surgical intervention.

## Conclusions

We report an incidental lesion suggestive of DNET identified during evaluation of acute headache and vomiting in a pediatric patient with concurrent GAS pharyngitis. In the absence of seizures or neurological deficits, conservative management with serial imaging was deemed appropriate in the case mentioned.

Headache is a frequent presentation in pediatric emergency departments and is usually due to benign causes; yet, the differential diagnosis remains wide and may occasionally include significant intracranial pathology. Although serious neurological etiologies are uncommon, the presence of red-flag features should prompt careful evaluation. DNET is a rare, benign glioneuronal tumor typically linked to epilepsy, so its incidental detection in a child without seizures or focal neurological deficits is unusual. This case emphasizes the need for clinicians to think beyond common differentials, correlate imaging with the overall clinical picture, and use a multidisciplinary approach to avoid both delayed recognition and unnecessary intervention.
